# Assembly, annotation and analysis of the chloroplast genome of the Algarrobo tree *Neltuma pallida* (subfamily: Caesalpinioideae)

**DOI:** 10.1186/s12870-023-04581-5

**Published:** 2023-11-16

**Authors:** Esteban Caycho, Renato La Torre, Gisella Orjeda

**Affiliations:** Laboratory of Genomics and Bioinformatics for Biodiversity, Faculty of Biological Sciences, Universidad Nacional Mayor de San Marcos, 15081 Lima, Peru

**Keywords:** *Neltuma pallida*, Chloroplast genome, Gene annotation, Repetitive sequences, Sequence comparison, Boundary shift

## Abstract

**Background:**

*Neltuma pallida* is a tree that grows in arid soils in northwestern Peru. As a predominant species of the Equatorial Dry Forest ecoregion, it holds significant economic and ecological value for both people and environment. Despite this, the species is severely threatened and there is a lack of genetic and genomic research, hindering the proposal of evidence-based conservation strategies.

**Results:**

In this work, we conducted the assembly, annotation, analysis and comparison of the chloroplast genome of a *N. pallida* specimen with those of related species. The assembled chloroplast genome has a length of 162,381 bp with a typical quadripartite structure (LSC-IRA-SSC-IRB). The calculated GC content was 35.97%. However, this is variable between regions, with a higher GC content observed in the IRs. A total of 132 genes were annotated, of which 19 were duplicates and 22 contained at least one intron in their sequence. A substantial number of repetitive sequences of different types were identified in the assembled genome, predominantly tandem repeats (> 300). In particular, 142 microsatellites (SSR) markers were identified. The phylogenetic reconstruction showed that *N. pallida* grouped with the other *Neltuma* species and with *Prosopis cineraria*. The analysis of sequence divergence between the chloroplast genome sequences of *N. pallida, N. juliflora*, *P. farcta* and *Strombocarpa tamarugo* revealed a high degree of similarity.

**Conclusions:**

The *N. pallida* chloroplast genome was found to be similar to those of closely related species. With a size of 162,831 bp, it had the classical chloroplast quadripartite structure and GC content of 35.97%. Most of the 132 identified genes were protein-coding genes. Additionally, over 800 repetitive sequences were identified, including 142 SSR markers. In the phylogenetic analysis, *N. pallida* grouped with other *Neltuma* spp. and *P. cineraria*. Furthermore, *N. pallida* chloroplast was highly conserved when compared with genomes of closely related species. These findings can be of great potential for further diversity studies and genetic improvement of *N. pallida*.

**Supplementary Information:**

The online version contains supplementary material available at 10.1186/s12870-023-04581-5.

## Background

**Fig. 1:**
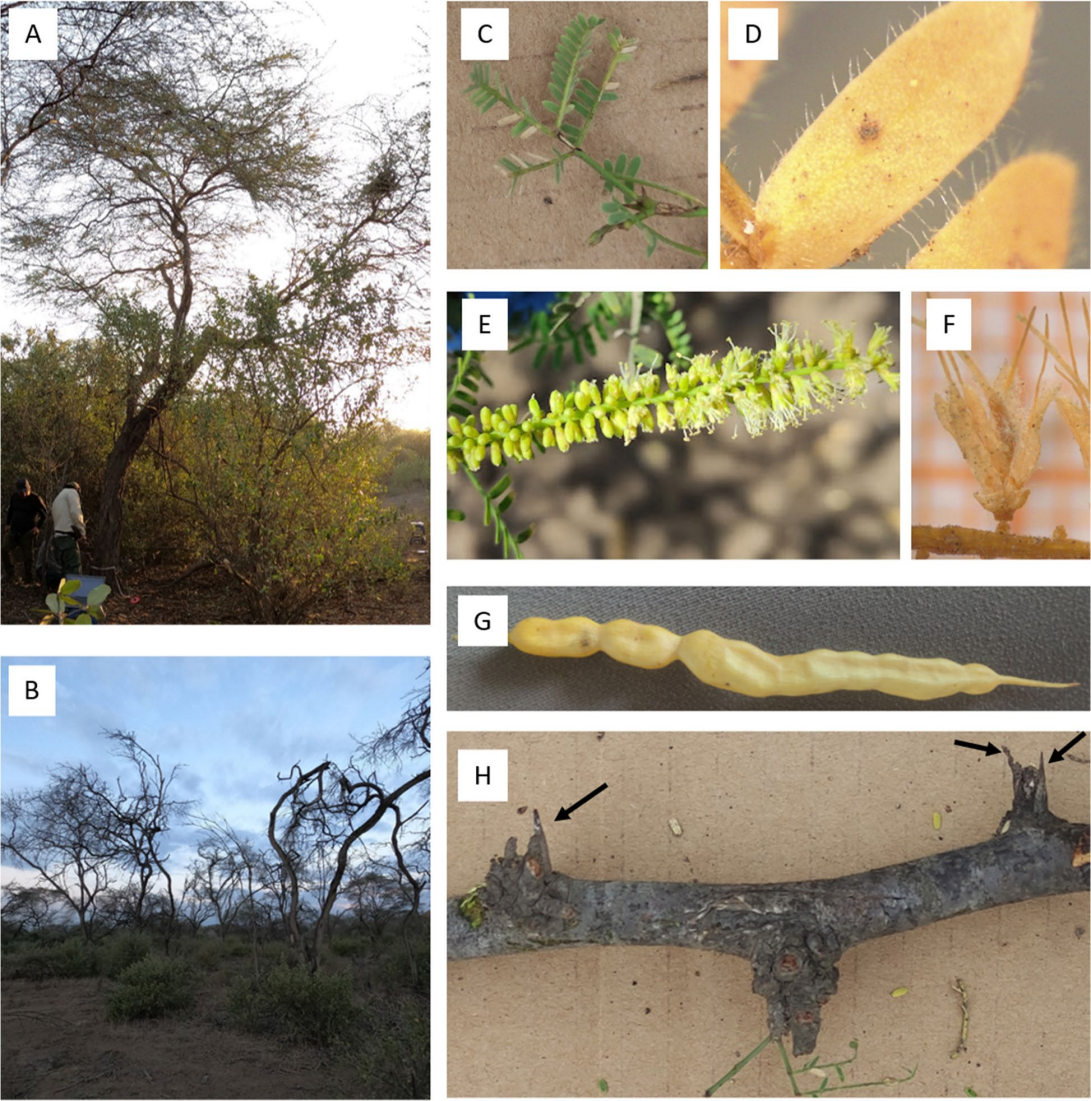
Pictures of *Neltuma pallida* trees and its main morphological descriptors. (A) Picture of the healthy tree whose DNA was used in this study. (B) Group of dead trees. (C) Bipinnate leaf. (D) Pubescence over a leaflet. (E) Inflorescence. (F) Single flower. (G) Mature pod. (H) Uninodal axillary spines (pointed by arrows)

The Algarrobo tree *Neltuma pallida* (Humb. & Bonpl. ex Willd.) Hughes & Lewis is a tree of 8 to 20 m in height, with small grayish-green leaves, uninodal axillary spines and yellow pod-shaped fruits [1, 2]. This species belongs to the genus *Neltuma* (Subfamily: Caesalpinioideae), which includes up to 43 potential species that are arboreal or shrubby, possess uninodal axillary spines, and are distributed in dry tropical and arid regions of America [2]. *Neltuma pallida* (Fig. 1), specifically, is native to arid regions of Colombia, Ecuador and Peru [1]. In Peru, Algarrobo trees grow in the Equatorial Dry Forest (3.45% of the country’s total area), an ecoregion located in the northern coastal regions such as La Libertad, Lambayeque, Piura and Tumbes [3–6], being one of the predominant species there.

Algarrobo trees hold significant economic and ecological importance in the South American countries, where they thrive [7, 8]. The species is known as “multipurpose”, offering a range of benefits to local communities [9]. The most common uses of Algarrobo trees are for fuel, medicine, cattle feed, or construction material [10]. Moreover, Algarrobo trees play a crucial role in the maintenance of their environment [11]. They provide a physical barrier to prevent wind erosion, contribute to soil fertility, maintain a microclimate and facilitate bioremediation [12, 13].

Despite the economic and ecological importance of *Neltuma* spp., their populations in Peru, Argentina, Chile, Venezuela, and the Chaco region (which includes some departments of Bolivia, Brazil, Argentina, and Paraguay) are experiencing a severe decline [14–18]. Regarding Peru, a report by the Peruvian National Forest and Wildlife Inventory [19] shows that as much as 40% of *N. pallida* trees are standing dead and 9% can be found as stumps. Of the 51% that remain alive, 27% display mild to severe damage. The true cause of the high mortality rate of the Algarrobo tree population is still uncertain and likely complex. Some hypotheses contemplate abiotic factors such as climate change [20] and drought [21] in the northern coast of Peru. Other hypotheses include biotic factors, such as the increasing presence of phytophagous and sucking pest insects [17, 22], now thought to be an effect of the decrease of natural biological controllers due to an ecological imbalance after recent ENSO events, or viruses of the Closteroviridae family [6, 23].

The literature shows few genetic studies on *N. pallida*, and genomic studies have not yet been carried out. In 2022, the National Institute of Agrarian Innovation (INIA) of Peru, together with the National University of Frontera (Piura - Peru), started a plant breeding project to improve the Algarrobo in Piura, a department in northern Peru [Arbizu, pers. comm]. Also, a project for molecular delimitation of *Neltuma* species has recently begun in our laboratory [Rivas M. pers. comm].

Some other works that have been published about the species focus on knowing its phenotypic variability, especially its characteristics of agronomic importance [24–26]. On the other hand, there are some studies from more than a decade ago that have sought to characterize the species at the genetic level [6]. These use classical molecular markers [27, 28], study the ploidy of the species [29–31], or analyze a single nucleotide sequence [32]. The lack of information on such an important and currently endangered species is detrimental to the development of comprehensive conservation and improvement strategies. Thus, the decline of Algarrobo tree populations continues to affect its highly fragile ecosystems, such as the deserts and dry forests of many South American countries. This is expected to lead to a decrease in endemism and biodiversity, as well as harsher living conditions.

A first step in the development of genomic studies in plants is the assembly and annotation of the chloroplast genome. This represents a faster and simpler task than sequencing and analyzing the nuclear genome due to its size and level of complexity [33, 34]. Chloroplast genome sizes range from 120 to 160 Kb, most commonly with 100 to 130 genes [35, 36]. In addition, genomic data is valuable for diversity studies [37, 38], phylogenetic analyses [39, 40], genetic improvement [41, 42], and genetic engineering of the species and closely related species [43, 44].

The objective of this work is to assemble, annotate and analyze the chloroplast genome of *Neltuma pallida*, and to compare it with other chloroplast genomes of close species. In this study, we discovered the chloroplast genome content of the Algarrobo tree *N. pallida* (subfamily: Caesalpinioideae) through its assembly, annotation and structural analysis. We also performed a comparative analysis using the sequenced chloroplast genomes of other species *Prosopis* sensu *lato* (s.l.), now the genera *Neltuma*, *Strombocarpa* and *Prosopis* sensu stricto (s.s.), and made a phylogenetic reconstruction to identify the relationships of *N. pallida* within the clade.

## Results

### Assembly and annotation of the chloroplast genome of *Neltuma pallida*

We assembled the chloroplast genome and obtained a graphical file of the assembly and the genome sequence. The graphical file was used to examine the structure and sequencing depth of the assembled genome. The assembled *N. pallida* chloroplast genome (Genbank: OR178743) had a length of 162,381 bp and the classical quadripartite structure (Fig. S1): a long single copy sequence (LSC) of 91,805 bp (~ 56.54% of the genome), a short single copy sequence (SSC) of 18,748 bp (~ 11.55%), and two inverted repeat (IR) regions of 25,914 bp (~ 31.91%) (Table 1).

**Table 1:** Summary of chloroplast genome features of *N. pallida* and related species

Species	*Neltuma pallida*	*Neltuma juliflora*	*Prosopis farcta*	*Strombocarpa tamarugo*	*Acacia ligulata*
Size (bp)	162,381	163,237	162,900	161,575	174,233
Total GC content (%)	35.97	35.9	35.88	36	35.4
LSC (bp)	91,805	92,495	92,156	91,062	88,576
SSC (bp)	18,748	18,880	18,880	18,643	18,298
IRs (bp)	25,914	25,931	25,932	25,935	25,925
Protein-coding regions (bp)	78,834	78,421	–	–	–
tRNA coding regions (bp)	2938	2927	–	–	–
rRNA coding regions (bp)	9052	9052	–	–	–
Number of genes	132	132	127	127	133
Number of protein-coding genes (PCG)	85	85	82	82	88
Number of rRNA	8	8	8	8	8
Number of tRNA	39	39	37	37	37
Number of genes with introns	22	21	25	18	18

LSC: Large Single Copy Region, SSC: Small Single Copy Region, IRs: Inverted Repeat Regions.

An analysis of the assembled genome structure was performed by examining the nucleotide composition of each region (Table S1). The GC content (GC%) of the whole genome was 35.97%. LSC and SSC had lower GC content: 33.26 and 30.46%, respectively. The IRs presented a higher GC% than the other regions and the whole genome with 42.77%. Among the coding regions, rRNA coding regions (located in the IRs) had the highest GC% with 55.41%, followed by tRNA coding regions with 53.10%. The lowest GC content was found in the protein coding regions with 37.45%.

The annotation of the assembled genome was done using the chloroplast genome of *N. juliflora* as reference. A total of 132 genes were found in the genome (19 duplicated genes), consisting of 85 protein-coding genes, 39 tRNA-coding genes and 8 rRNA-coding genes. The 85 protein-coding genes correspond to 78,834 bp, the 39 tRNA-coding genes to 2938 bp, and the 8 rRNA-coding genes to 9052 bp (Table 2).

**Table 2:** Genes annotated in the *N. pallida* chloroplast genome grouped by category and functional group

Category	Functional group	Annotated genes
Transcription and translation	Large subunit of ribosomal proteins	*rpl*2”*, *rpl*14, *rpl*16*, *rpl*20, *rpl*23”, *rpl*32, *rpl*33, *rpl*36
	Small subunit of ribosomal proteins	*rps*2, *rps*3, *rps*4, *rps*7”, *rps*8, *rps*11, *rps*12”*, *rps*14, *rps*15, *rps*16*, *rps*18, *rps*19
	DNA dependent RNA polymerase	*rpo*A, *rpo*B, *rpo*C1*, *rpo*C2
	Translation	*inf*A
	rRNA	*rrn*4.5″, *rrn*5”, *rrn*16”, *rrn*23”
	tRNA	*trn*A-UGC”*, *trn*C-GCA, *trn*D-GUC, *trn*E-UUC, *trn*F-GAA, *trn*G-GCC*, *trn*G-UCC, *trn*H-GUG, *trn*I-CAU”, *trn*I-GAU”*, *trn*K-UUU*, *trn*L-CAA”, *trn*L-UAA*, *trn*L-UAG, *trn*fM-CAU, *trn*M-CAU”, *trn*N-GUU”, *trn*P-GGG, *trn*P-UGG, *trn*Q-UUG, *trn*R-ACG”, *trn*R-UCU, *trn*S-GCU, *trn*S-GGA, *trn*S-UGA, *trn*T-GGU, *trn*T-UGU, *trn*V-GAC”, *trn*V-UAC*, *trn*W-CCA, *trn*Y-GUA
Photosynthesis	Photosystem I	*psa*A, *psa*B, *psa*C, *psa*I, *psa*J
	Photosystem II	*psb*A, *psb*B, *psb*C, *psb*D, *psb*E, *psb*F, *psb*H, *psb*I, *psb*J, *psb*K, *psb*L, *psb*M, *psb*N, *psb*T, *psb*Z
	NADH dehydrogenase	*ndh*A*, *ndh*B”*, *ndh*C, *ndh*D, *ndh*E, *ndh*F, *ndh*G, *ndh*H, *ndh*I, *ndh*J, *ndh*K
	Cytochrome b6/f complex	*pet*A, *pet*B*, *pet*D*, *pet*G, *pet*L, *pet*N
	ATP synthase	*atp*A, *atp*B, *atp*E, *atp*F*, *atp*H, *atp*I
	Rubisco	*rbc*L
Other genes	Maturase	*mat*K
	Protease	*clp*P*
	Envelope membrane protein	*cem*A
	Subunit Acetyl-CoA-Carboxylase	*acc*D
	c-type cytochrome synthesis gene	*ccs*A
Unknown	Conserved Open reading frames	*ycf*1”, *ycf*2”, *ycf*3*, *ycf*4

”Duplicated genes, * Genes with introns.

**Fig. 2:**
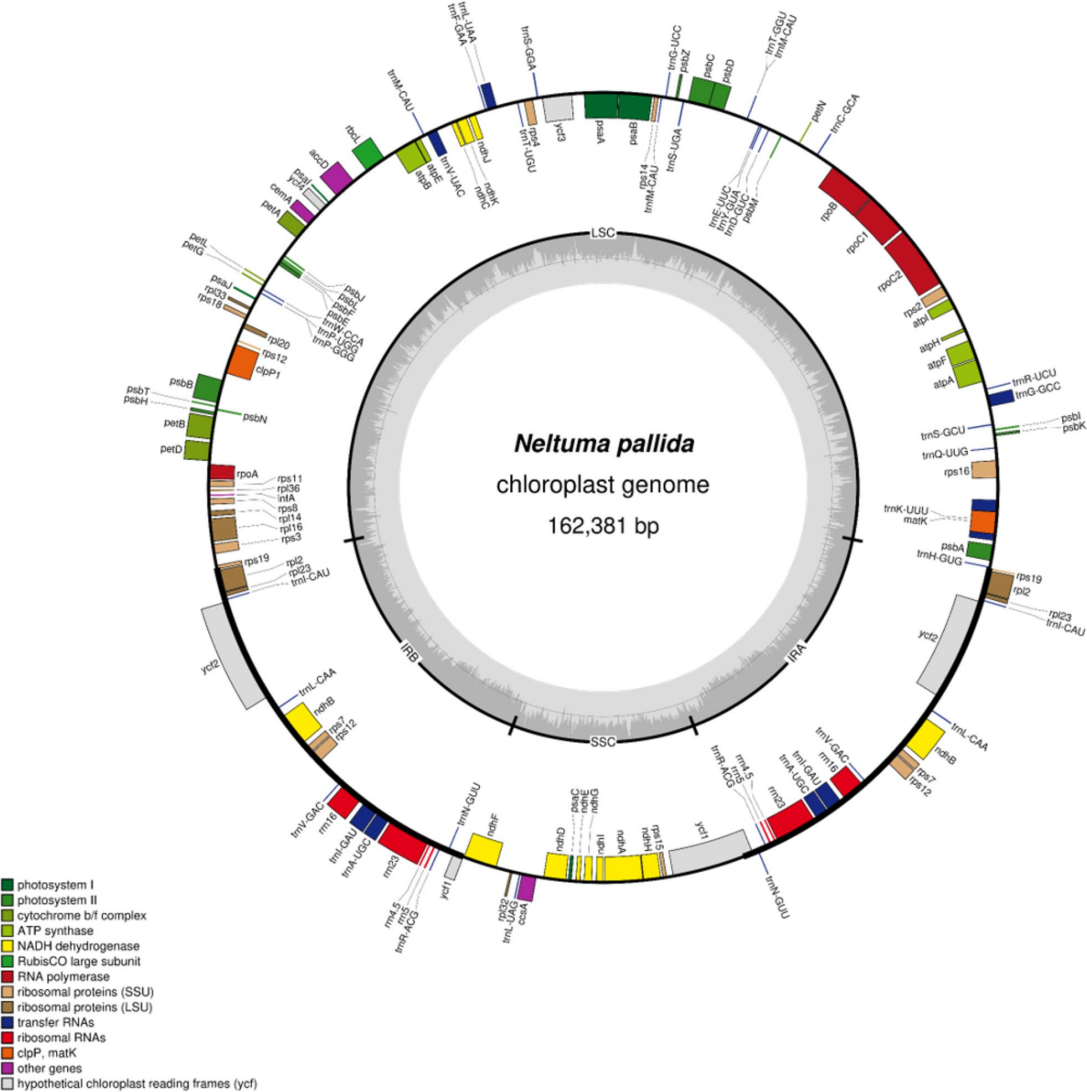
Genome map of *Neltuma pallida* chloroplast genome. It shows its four genomic regions (LSC, SSC, IRA and IRB). Genes located in the inside of the circle are transcribed clockwise, while those located in the outside are transcribed in the opposite direction. Genes are grouped according to their functional group by color codes. The inner circle exhibits de GC content (dark gray) and the AT content (light gray)

The annotated genes included the following (Fig. 2): 12 small ribosomal proteins (*rps*), 8 large ribosomal proteins (*rpl*), 4 DNA-dependent RNA polymerases (*rpo*), 4 different rRNA coding genes (*rrn*), 31 different tRNA coding genes (*trn*), 5 photosystem I proteins (*psa*), 15 photosystem II proteins (*psb*), 11 NADH dehydrogenase proteins (*ndh*), 6 cytochrome b6/f complex proteins (*pet*), 6 ATP synthase complex proteins (*atp*), the major subunit of ribulose-1,5-bisphosphate carboxylase/oxygenase (*rbc*L), maturase K (*mat*K), the proteolytic subunit of ATP-dependent Clp protease (*clp*P), membrane envelope protein (*cem*A), beta subunit of acetyl-CoA carboxylase (*acc*D), cytochrome C biogenesis protein (*ccs*A), 4 hypothetical proteins of unknown function (*ycf*), and translation initiation factor 1 (*inf*A). This makes a total of 113 different genes, 18 of which present introns (16 have one intron and 2 have two introns) (Table 3).

**Table 3:** Genes containing introns within the chloroplast genome of *N. pallida* and the length of their respective exons

Gene	Region	Exon I (bp)	Intron I (bp)	Exon II (bp)	Intron II (bp)	Exon III (bp)
*atp*F	LSC	146	726	407		
*clp*P	LSC	69	789	291	641	228
*ndh*A	SSC	553	1450	539		
*ndh*B	IR	777	685	756		
*pet*B	LSC	6	814	642		
*pet*D	LSC	8	720	475		
*rpl*2	IR	393	662	435		
*rpl*16	LSC	9	1129	399		
*rpo*C1	LSC	432	801	1617		
*rps*12	LSC-IR	114		232	536	26
*rps*16	LSC	40	883	245		
*trn*A-UGC	IR	38	802	35		
*trn*G-GCC	LSC	23	702	49		
*trn*I-GAU	IR	42	948	35		
*trn*K-UUU	LSC	37	2611	29		
*trn*L-UAA	LSC	37	536	50		
*trn*V-UAC	LSC	39	621	35		
*ycf*3	LSC	126	728	228	739	153

**Fig. 3:**
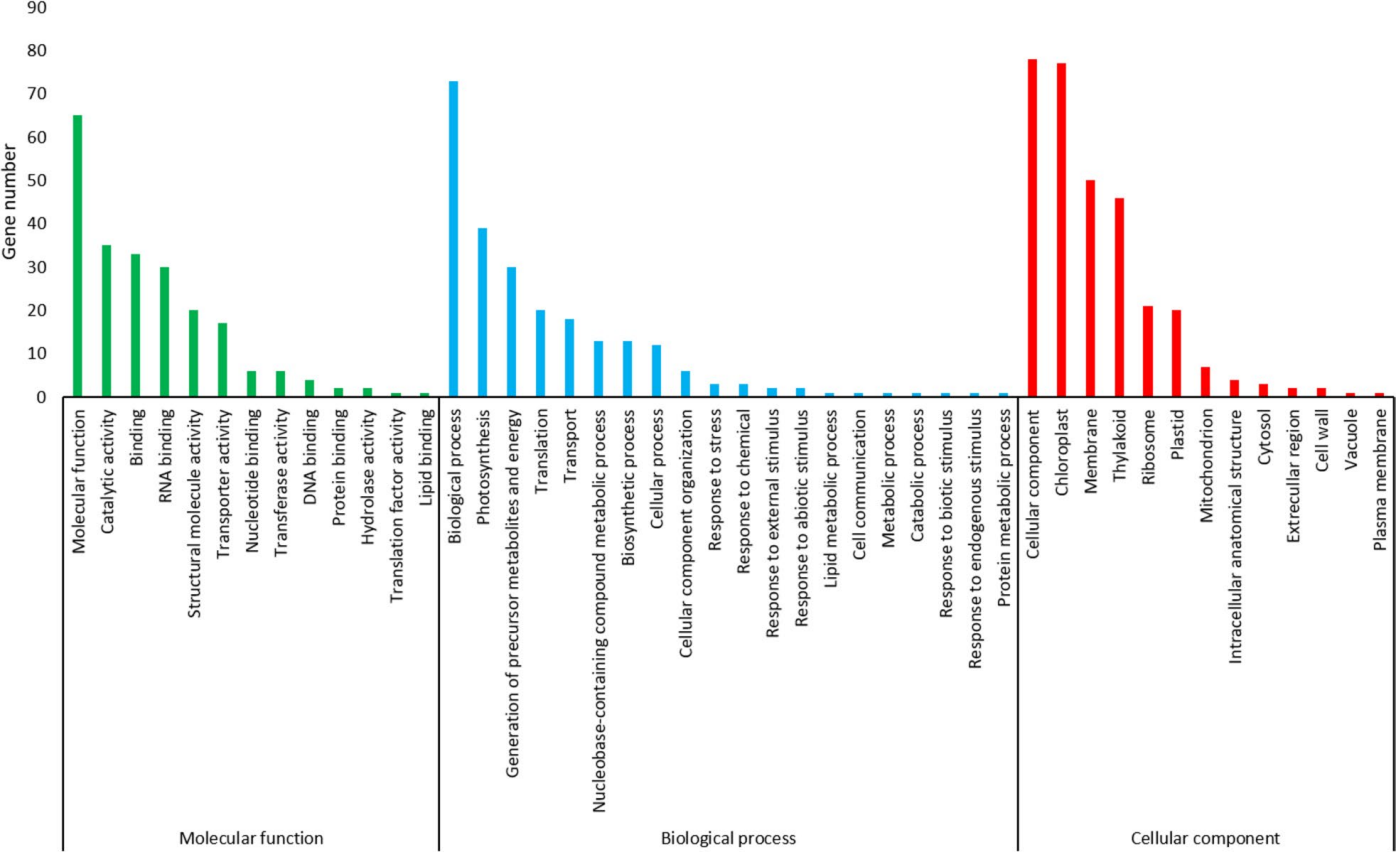
Histogram of *N. pallida* chloroplast genes distribution in Gene Ontology (GO) terms

Functional annotation was conducted on the protein-coding genes sequences, to identify metabolic pathways and processes. Based on information obtained from the KEGG database, these genes belong to four primary classes (metabolism, genetic information processing, cellular processes and organ systems) (Table S2). Metabolism is the class with the highest number of genes, with energy metabolism being particularly prominent. According to the UniProt database, most of the genes are involved in biological processes, photosynthesis, and generation of metabolite precursors and energy (Fig. 3). Additionally, a large number of genes relate to cellular components and the chloroplast.

### Codon usage analysis

**Fig. 4:**
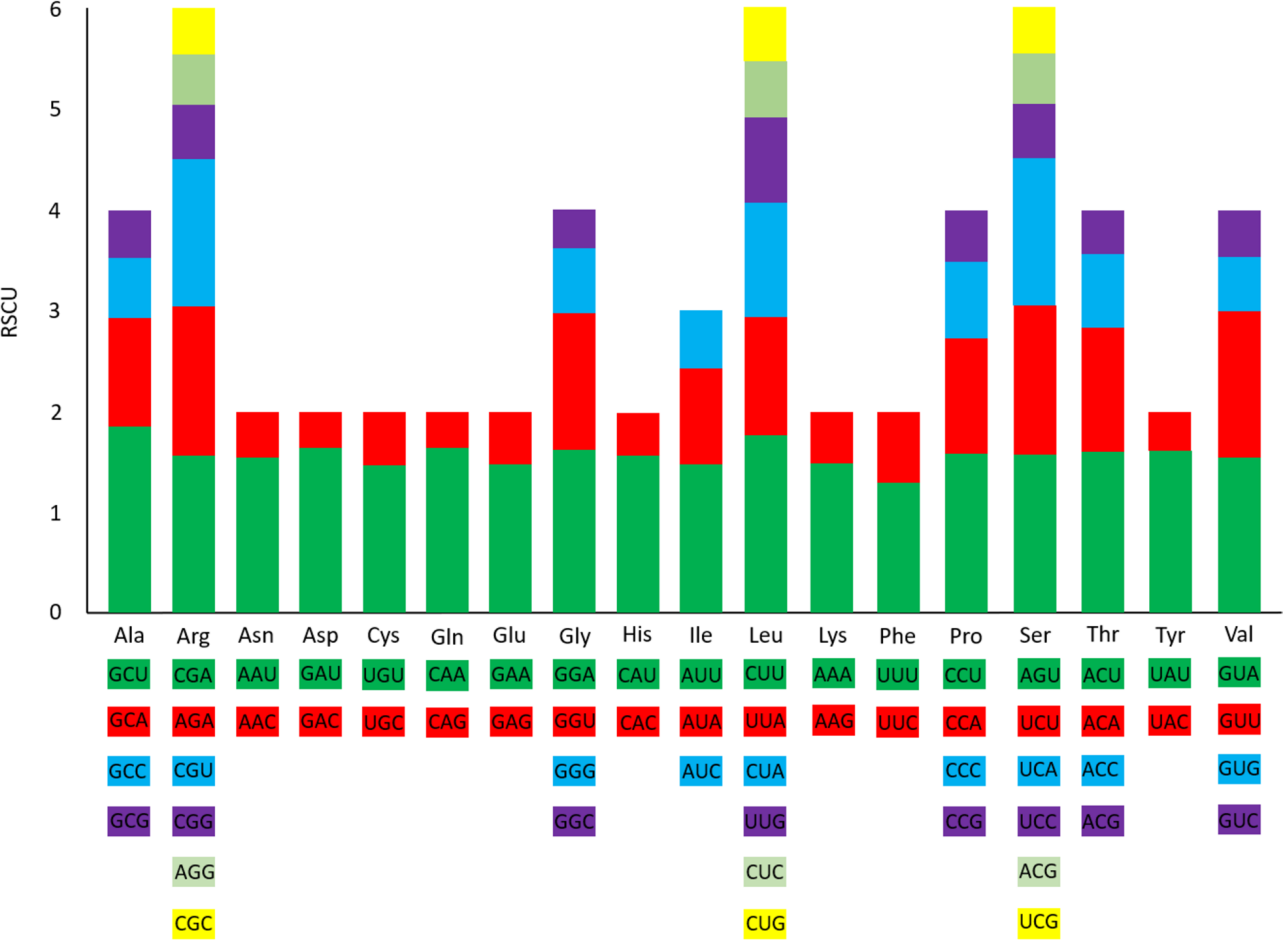
RSCU values of amino acids in 85 protein-coding genes of the *Neltuma pallida* chloroplast genome

Codon frequency (Table S3) and the Relative Synonymous Codon Usage (RSCU) were calculated for the whole exome (protein-coding regions). The most frequently used codon in the genome was AAU, which codes for Isoleucine (*n* = 1144), followed by AAA, which codes for Lysine (*n* = 1069). At the other extreme, the least used codons were the three types of STOP codons UGA (*n* = 17), UAG (*n* = 18) and UAA (*n* = 50). The less frequent amino acid-coding codons were UGC, coding for Cysteine (*n* = 85), and CGC, coding for Arginine (*n* = 103). Analyzing the RSCU values, it could be seen that, for each amino acid, half of the codons were used with a higher relative frequency than the other half. Also, the preferred codons were A or T/U ending codons (Fig. 4).

### Identification of repetitive sequences

**Fig. 5:**
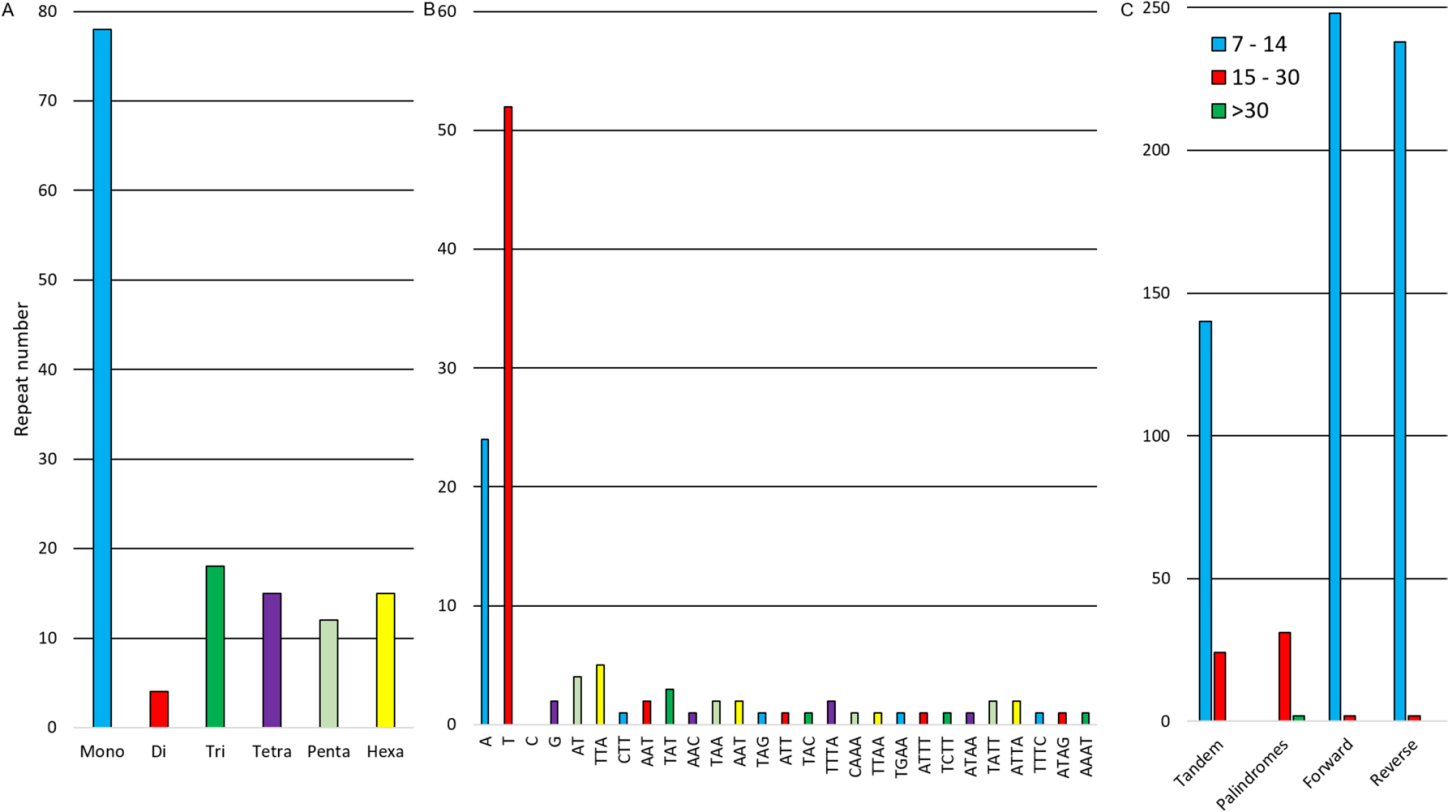
Repetitive sequences frequency in the *Neltuma pallida* chloroplast genome. (A) Total SSR frequency. (B) SSR frequency according to its repeat motif. (C) Tandem, palindrome, forward and reverse repeats frequency

We searched for repetitive sequences in the genome. The tandem repeats were identified as microsatellites (SSRs) and tandem repeats in general. A total of 142 SSRs were identified (Fig. 5A), with the most abundant repeats being mononucleotide repeats (*n* = 78), mostly A/T mononucleotides (Fig. 5B). For the remaining SSR (di- to hexanucleotide repeats), between 4 and 18 repeats were found. Tandem repeats larger than hexanucleotides were also identified, with 164 repeats found (Fig. 5C). Thus, a total of 306 tandem repeats were found.

The rest of the repeats were palindromes, direct repeats and inverted repeats. In the genome, 33 palindromes, 250 direct repeats and 240 inverted repeats were found, most of them having between 15 and 29 nucleotides (Fig. 5C).

### Phylogenomic relationships of *Neltuma pallida*

**Fig. 6:**
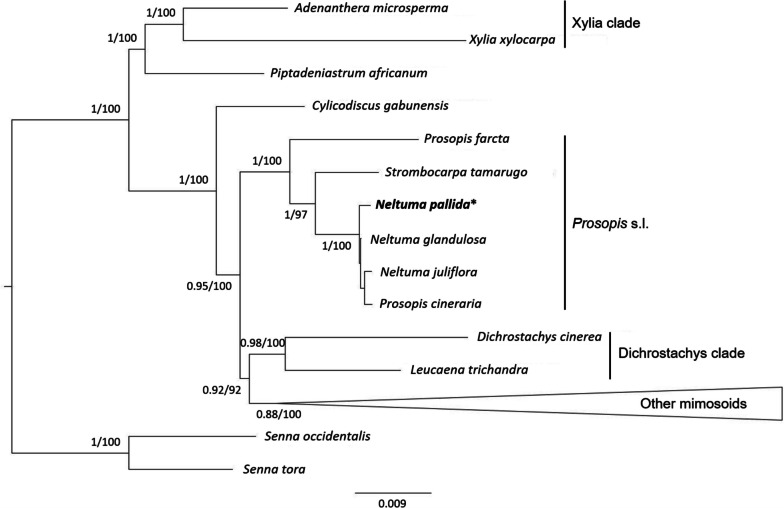
Phylogenomic tree of *N. pallida* within subfamily Caesalpinioideae. The whole chloroplast genomes were used for this reconstruction. The methods employed were Bayesian Inference and Maximum Likelihood, and their statistics values are represented by the numbers on the left of each node. *N. pallida* position in the tree is highlighted with an asterisk (*)

A phylogeny of the subfamily Caesalpinioideae was constructed using the chloroplast genomes of 30 previously published species to determine the position of *N. pallida* in the clade. We found that the genus *Neltuma* was not recovered as a monophyletic group because *N. pallida* was grouped with the other species of *Neltuma* spp. and *P. cineraria* (Fig. 6). Beside this, the genus *Neltuma* is grouped with the other genera of *Prosopis* s.l. used in this analysis: *Prosopis* s.s. and *Strombocarpa*. *Prosopis* s.l. also shows closeness to *Cylicodiscus gabunensis* and the Dichrostachys clade.

### Sequence divergence analysis

**Fig. 7:**
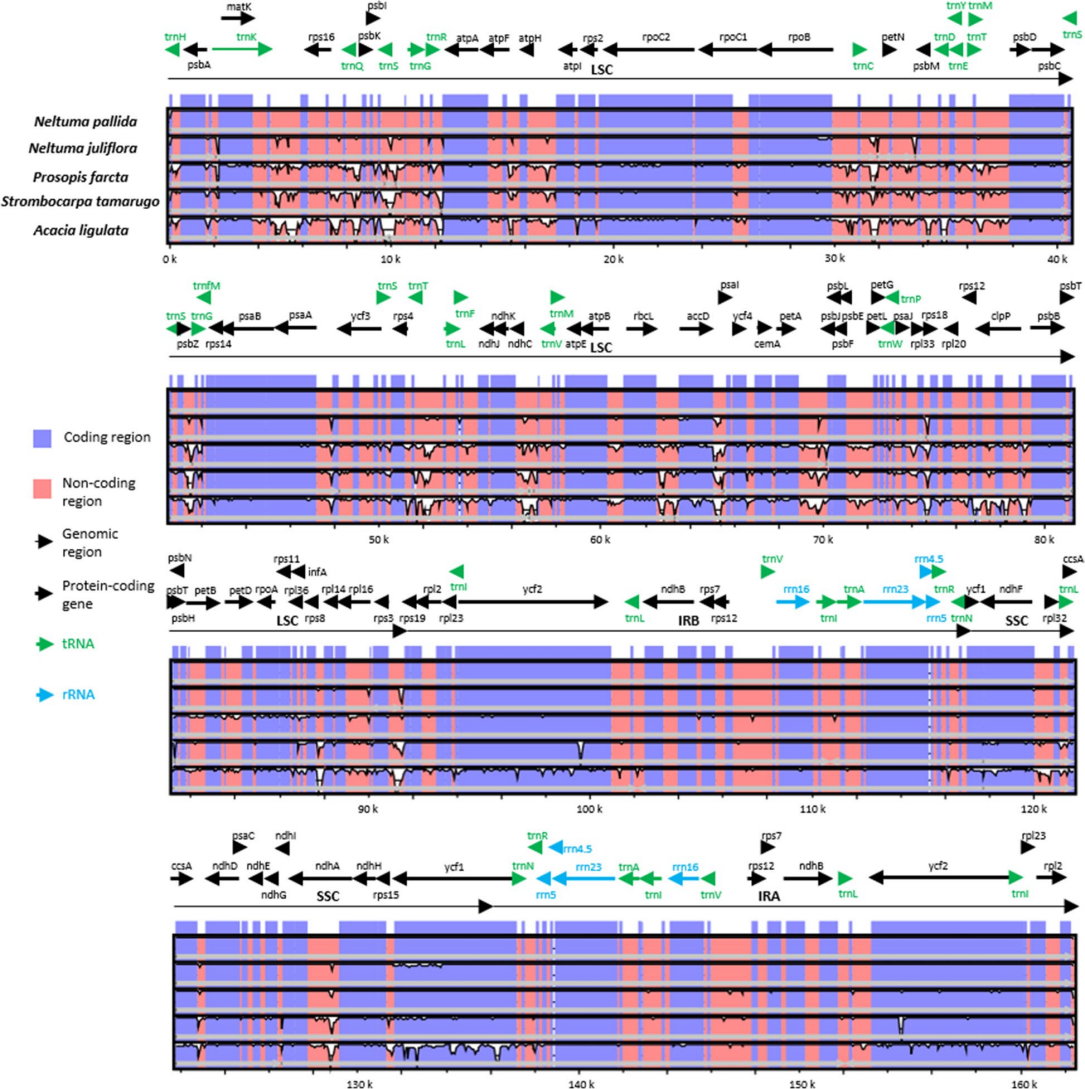
Graphical representation of the alignment of the chloroplast genomes of *N. pallida* and related species. *N. juliflora*, *P. farcta*, *S. tamarugo* and *A. ligulata* are used for comparison. The graph shows the level of identity (50–100%, Y-axis) along the genomes (X-axis) taking as reference the chloroplast genome of *Neltuma pallida*

Divergence between the sequences of *N. juliflora*, *P. farcta*, *S. tamarugo* and *A. ligulata* with *N. pallida* was calculated (Fig. 7). The lowest divergence was observed with *N. juliflora*, while the highest divergence was observed with *A. ligulata*. When looking at the genomic regions, it was found that the most conserved regions are the IRs, regardless of the species to which they are compared. The opposite was determined for the single copy regions (LSC and SSC), where the greatest divergence between sequences was found.

As expected, non-coding regions show the highest divergence, highlighting some intergenic regions such as *trn*K-*rps*16, *trn*S-*trn*G, *trn*C-*pet*N, *psb*Z-*trn*G, *trn*T-*trn*L, *rbc*L-*acc*D, *acc*D-*ycf*4, *rps*8-*rpl*14 and *rps*3-*rps*19 in LSC or *ccs*A-*ndh*D and *rps*15-*ycf*1 in SSC. Also, some intronic regions show a higher degree of divergence such as in *trn*K, *rps*16, *atp*F, *rpo*C1, *rpl*2, *rpl*16 and *clp*P in LSC or *pet*B, *pet*D and *ndh*A in SSC.

In the coding regions the divergence was smaller, especially when compared to other *Prosopis* s.l. species. The genes with the greatest difference in their coding sequences are *rpo*C2, *rpo*B, *acc*D, *cem*A, *pet*A, *rpl*20, *ycf*2, *ndh*F and *ycf*1. There are also genes that show divergence only in one of the species of this group, this is the case of *atp*A, *psb*C, *psa*A and *psb*B in *P. farcta*.

### Genetic distance of coding sequences analysis

**Fig. 8:**
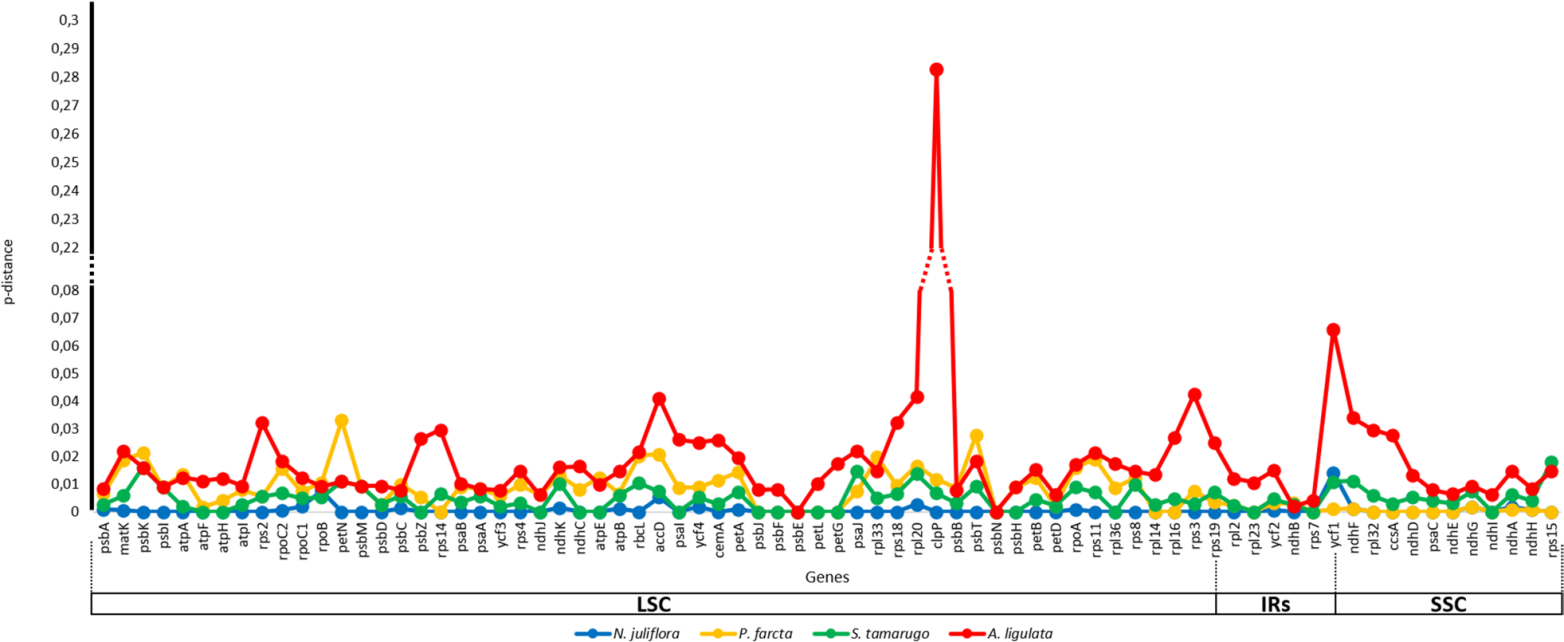
Pairwise distance of 74 protein-coding genes from *N. pallida* chloroplast genome with homologs. The gene’s homologs were obtained from *N. juliflora*, *P. farcta*, *S. tamarugo* and *A. ligulata* chloroplast genomes

Genetic distance was determined using the sequences of 74 chloroplast genes taken pairwise with the p-distance algorithm. For this purpose, the genome of *N. pallida* was compared as a reference with those of *N. juliflora*, *P. farcta*, *S. tamarugo* and *A. ligulata* (Fig. 8). As expected, the highest average distance was found between *N. pallida* and *A. ligulata* (0.0200), while the lowest was found between *N. pallida* and *N. juliflora* (0.0007).

The 10 genes with the largest genetic distance to *N. pallida* sequences were *clp*P (0.0754), *ycf*1 (0.0231), *rpl*20 (0.0188), *acc*D (0.0187), *psb*T (0.0139), *pet*N (0.0139), *psb*K (0.0134), *rps*3 (0.0133), *rbc*L (0.0131) and *rps*18 (0.0122).

### Boundary between regions

**Fig. 9:**
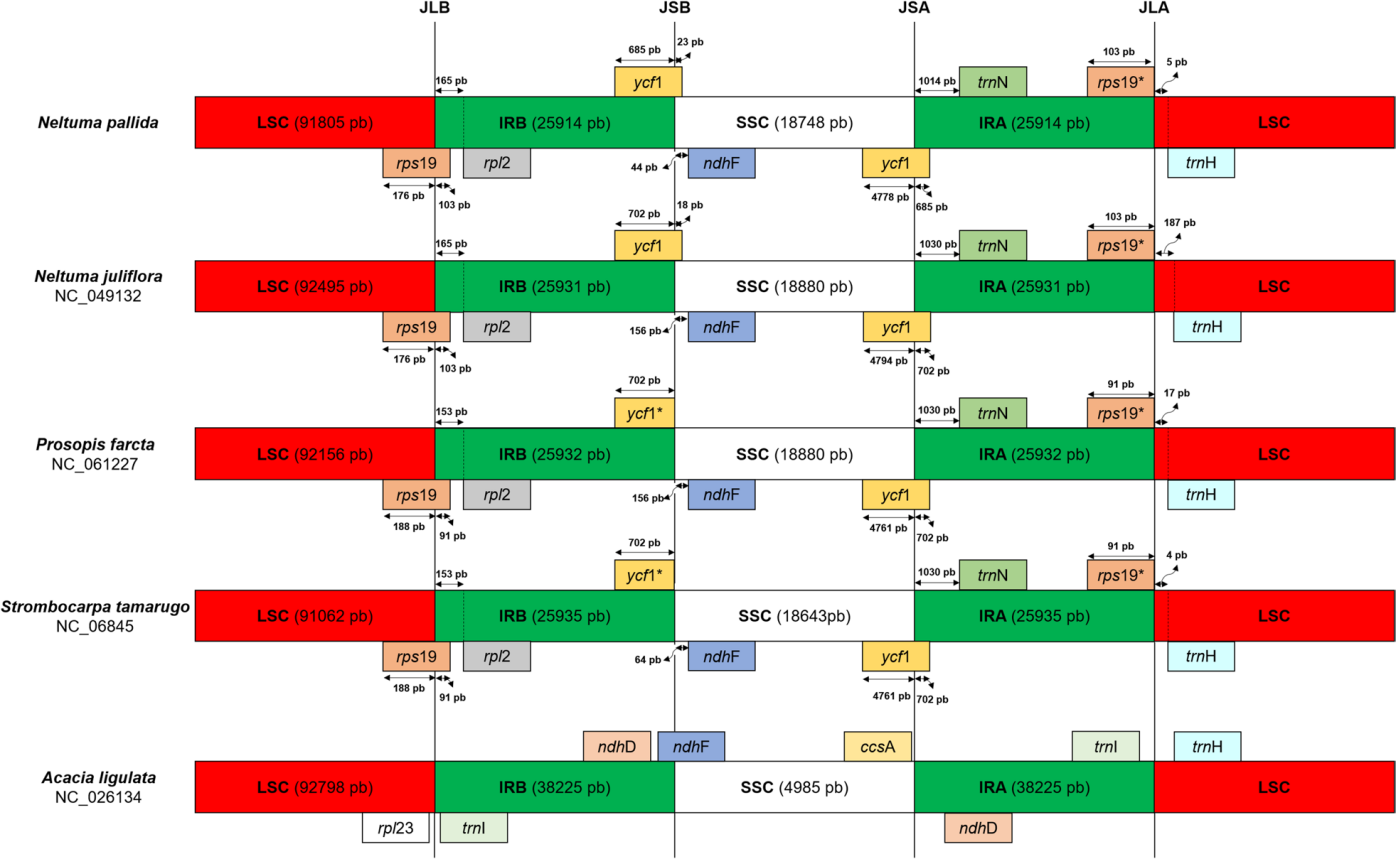
Comparison of boundaries between the regions of the *N. pallida* chloroplast genome with related species. The species used for this comparison were *N. juliflora*, *P. farcta*, *S. tamarugo* and *A. ligulata*. The genomes are represented as split bars in each region. The boxes above and below the bars are representations of the genes. The arrows indicate the distance in bp between the ends of the genes with the boundaries closest to this one. These representations are not proportional to sequence lengths. *rps*19* (non-coding) found in IRA is a portion of the complete rps19 gene found in IRB

The expansion and contraction of the LSC, IRB, SSC, and IRA of the *N. pallida* chloroplast genome were analyzed by examining the distance between their boundaries with their nearest genes (Fig. 9), and comparing these distances with related species.

In all *Prosopis* s.l. species, the genes closest to the boundary between the LSC and IRB regions (called JLB) were *rps*19 and *rpl*2. In all cases, the *rps*19 gene overlaps with both regions, and *rpl*2 falls within the IRB region. When comparing the distances of *rpl*2 to JLB between *Neltuma* spp. and *Prosopis* s.l. species, a contraction (12 bp) in IRB and an expansion in LSC are found.

Moving to the next boundary, between IRB and SSC (called JSB), in all *Prosopis* s.l. species, the closest genes are *ycf*1 and *ndh*F. When comparing *N. pallida* with the rest of the *Prosopis* s.l. species, we found a 17 bp contraction in IRB and a 20–112 bp contraction in SSC.

At the boundary between SSC and IRA (called JSA), the closest genes for *Prosopis* s.l. species were the other copy of *ycf*1 and *trn*N. When comparing the distance between this boundary and the closest genes, it was found that in the *N. pallida* SSC, there was a contraction of 6 bp with respect to the distance found in *N. juliflora* and an expansion of 17 bp with respect to the distance found in *P. farcta* and *S. tamarugo*. On the other hand, in *N. pallida* IRA, a contraction of 16 bp was observed with respect to the rest of the *Prosopis* s.l. species.

Finally, near the boundary between IRA and LSC (called JLA), the *rps*19 pseudogene and the *trn*H gene were found in *Prosopis* s.l. species. In the IRAs of *Prosopis* s.l., no differences were found between species, starting from the *rps*19 pseudogene in the IRA and reaching the boundary with LSC. The opposite case was observed in the LSC, when comparing *N. pallida* with the rest of the *Prosopis* s.l. species, a contraction of 12 and 182 bp was found with respect to *P. farcta* and *N. juliflora*, respectively. With respect to *S. tamarugo,* an expansion of only 1 bp difference was found.

Different distances were observed between boundaries and their nearest genes, these differences were small among species within *Prosopis* s.l. We also analyzed the case of *A. ligulata* and found two differences. First, the boundaries between regions in the chloroplast genome of *A. ligulata* were at completely different positions than in the *Prosopis* s.l. species used in this analysis. Second, the genes closest to the boundaries were different.

## Discussion

The total genomic DNA of *N. pallida* was sequenced using Illumina technology, and its chloroplast genome sequences were extracted and assembled with GetOrganelle. This genome was annotated and compared with chloroplast genomes of other species of *Prosopis* s.l. The assembled chloroplast genome of *N. pallida* was 162,381 bp (162.4 Kb) long, with a classical circular quadripartite structure: two inverted repeats (IRA and IRB), a short single-copy region (SSC) and a long single-copy region (LSC) (Fig. 2; Table 1). The same quadripartite structure and similar chloroplast genome sizes have been reported in species of the genera *Prosopis*, *Neltuma* and *Strombocarpa* (161.5–163.7 kb) [45–47]. The same has been found in other mimosoid species [48, 49] and legumes [45]. In general, chloroplast genomic regions conserve similar sizes among closely related species, as in *Prosopis* s.l. However, some mimosoid species present regions of different length. This is the case for species of the Inga clade and *Albizia* spp. [50, 51], which exhibit expansions in their IRs and reductions in their SSCs, generating longer genomes. Our results agree with some studies mentioning that differences in chloroplast genome size are explained by variations in the length of single copy regions, LSC and SSC [46, 52]. Comparing the size differences between IRs and single copy regions in *Prosopis* s.l., it was found that differences between LSCs and SSCs were always larger than those between IRs.

In the chloroplast genome of *N. pallida*, 132 genes were identified and the 19 genes located in the IRs were duplicated (Fig. 2, Table 2). The genes in the IRs were also duplicated in the chloroplast genomes of *P. cineraria* and *N. juliflora* [46]. Duplication of genes in the IRs is common as it has been observed in other mimosoid species, including those with longer IRs. The difference with these species is that their IRs contain a larger number of genes [50, 51]. The same 19 duplicated genes have also been identified as duplicates in other legumes that are not mimosoids [53]. The duplication of the IRs genes in the chloroplast is common regardless of the length of the region and the phylogeny.

Gene prediction annotation identified 15 genes with one intron and 3 with two introns (*clp*P, *rps*12 and *ycf*3) (Table 3). Similarly, to other chloroplast genomes, *rps*12 was unevenly distributed in LSC (first exon) and IRB (second and third exons) [54, 55]. Additionally, in the chloroplast genome of *N. pallida* we identified genes that were absent in related species. For instance, we found *trn*G-GCC, which is not present in *P. cineraria* or in *N. juliflora* chloroplast genomes, while it is in *N. glandulosa* [46]. The gene coding for the transcription initiator factor, infA, was also annotated in *N. pallida*, as it presents an open reading frame (ORF). This ORF is also present in the chloroplast genomes of *Prosopis*, *Strombocarpa* or *Neltuma*, but has not been annotated in those genomes. This gene is either absent or present only as a pseudogene in many legume species [53, 56, 57]. The ORF of this gene has been identified in various chloroplast genomes of other legumes, such as *Albizia julibrissin* and *Lespedeza* spp. [51, 58]. It has been proposed that *inf*A is a gene that has been lost multiple times from chloroplast genomes during the evolutionary history of angiosperms, and has subsequently been transferred to the nuclear genome of plants [59]. Additionally, there is the interesting case of a *rps*19 segment (103 bp) which is located in IRA as a pseudogene. This rps19 pseudogene has been discovered in all the other species of *Prosopis* s.l., with incomplete ORFs [46]. The same pseudogene has been found in non-legumes species, such as *Cerasus humilis* (family: Rosaceae) [60] and *Garcinia paucinervis* (family: Clusiaceae) [61].

The GC content (GC%) of the whole chloroplast genome of *N. pallida* was determined to be 35.97% (Table 1). Comparable values have been observed in the chloroplast genomes of *P. cineraria* and *N. juliflora* [46]. These values are typical for most sequenced chloroplast genomes, with GC content around 36.2%, except for certain taxonomic groups like *Selaginella* spp. (family Selaginellaceae), with 54.8% [62, 63]. Lower GC% values were found in single copy regions (33.26 and 30.46% for LSC and SSC, respectively), but GC% was higher in IRs (42.77%). Previous studies have suggested that this increase in GC% value in IRs is due to the presence of rRNA and tRNA genes [46, 64, 65]. Our results support this presumption as the GC% of rRNA and tRNA genes was calculated to be higher than 53%. It is noteworthy that the high GC% found in IRs aids in their preservation, as it has been shown to decelerate the rate of nucleotide substitution [66].

Regarding codon usage in protein-coding genes, a clear preference was observed for those ending in A or T/U, having RSCU values higher than 1. This pattern was observed in *Albizia julibrissin* [51] and *Stryphnodendron adstringens* [48], both species from the subfamily Caesalpinioideae, and in more distant species such as *Salix floderusii* [67] or *Chrysosplenium* spp. [68]. Also, Duan et al. [69] found a preference for the use of codons ending in A or T/U. They determined that there is a selective pressure favoring the use of these codons. This evolutionary pressure has only been detected in the third position of the codon, but not in the rest of the chloroplast genome. This positive selective pressure facilitates the expression efficiency and conservation of highly important genes [70], which may explain the observed conservation in the third position of codons.

We identified 142 microsatellites (SSRs) in the *N. pallida* chloroplast genome, with mononucleotide repeats being the most abundant. This is similar to the findings of Asaf et al. [46] in the chloroplast genomes of *P. cineraria* and *N. juliflora, although* they found a lower number of SSRs and did not find penta- or hexanucleotides in those genomes. We also identified more repeats of all types compared to other chloroplast genomes of *Prosopis*, *Strombocarpa* or *Neltuma*. This could be attributed to the use of distinct tools to detect repetitive sequences. As noted by Das & Ghosh [71] dedicated software programs for the identification of repetitive sequences commonly produce varying outcomes based on the algorithm utilized. Despite the challenges that arise from algorithmic disparity, it is still feasible to draw general comparisons, such as the higher number of repetitive mononucleotides among SSRs in the chloroplast genomes of distant species [39, 72, 73]. There is a need to establish a standardized methodology for the identification of repetitive sequences in the chloroplast genomes. This will allow for more reliable comparisons. Repetitive sequences analysis within chloroplast genomes is of high importance as they serve to study genetic diversity and phylogeny through molecular markers development, as emphasized in previous work [72, 74]. Likewise, chloroplast genomes exhibit considerable variation in their repetitive sequences’ number of tandem repeats, which may be useful for detecting population-level polymorphisms [75].

The chloroplast genome sequence is a powerful tool frequently utilized to elucidate phylogenetic relationships [76, 77]. However, cases of discordance between chloroplast and nuclear phylogenies can also be found [78, 79]. This is why information from both sources should be used concurrently when possible. In this work, *N. pallida* formed a subclade with *N. juliflora*, *N. glandulosa and P. cineraria*. The subclade with these four species, formerly included in the *Prosopis* genus, was reported by Asaf et al. [46], as a monophyletic group. This differs completely with Hughes et al. [2] whose recent results support that *Prosopis* s.l. is polyphyletic. Their research included about 1000 nuclear genes sequenced by Ringelberg et al. [80] in combination with morphological characters. The author suggested separating *Prosopis* s.l. into 4 different genera, placing *N. pallida*, *N. juliflora* and *N. glandulosa* in the genus *Neltuma*. On the other hand, *P. cineraria* and *P. farcta*, remained as species of the genus *Prosopis* s.s. *P. cineraria* and *P. farcta* originated and can be found in western Asia [81–83], while *N. pallida*, *N. juliflora* and *N. glandulosa* are native to the Americas [2, 7]. Our outcomes diverged from Hughes et al.’s [2] research since we found that *P. cineraria* is distanced from *P. farcta* in our phylogenetic tree (Fig. 6). Literature reports further cases of incongruent phylogeny between nuclear and chloroplast information [84–89]. In all these cases, incomplete lineage delimitation or introgression/hybridization was found to have influenced the discordances. Both options are feasible in certain Caesalpinioideae species, such as *Prosopis cineraria*. There is also evidence of interspecific and intergeneric hybridization in *Prosopis* s.l. [90, 91]. Nevertheless, due to the lack of reference chloroplast genome sequences for other Caesalpinioideae species, they could not be included in our analysis, which hinders the draw of conclusion.

When analyzing the divergence between chloroplast genome sequences among species of *Prosopis* s.l., a high level general of conservation was observed. However, it was also found: first, that non-coding regions, including intergenic sequences and introns, display the lowest similarity; second, that LSC and SSC exhibit the lowest identity, as has been seen across different taxonomic groups [92–94]; and lastly, that IRs are highly conserved, which aligns with the importance of the biological functions they serve in terms of genomic stabilization and gene conservation [95]. Thus, a single mutation in IRs may cause structural and sequence changes at many other sites in chloroplast genomes [96–98]. Another interesting point when comparing chloroplast sequences of *Prosopis* s.l. is that variations were only detected in 9 genes. A similar finding was made by Asaf et al. [46] in their comparison of *N. juliflora* and *P. cineraria*, with *N. glandulosa*, showing divergence in 5 of these 9 genes. This may indicate that this group’s chloroplast genomes are particularly susceptible to mutations in these genes. Despite the observed variability, the genetic distances were minimal. The *clp*P gene exhibited the most significant variability (0.0754). This gene, which encodes a subunit of the ATP-dependent chloroplast protease, has been reported to show a high rate of amino acid substitution, which is associated with protein structure variability [99]. The second gene with the highest genetic distance was *ycf*1 (0.0231). Some studies highlight that *ycf*1, in combination with the intergenic region between it and the *ndh*F gene, can be applied as a barcode for land plants [100, 101], indicating its high diversity level.

Boundary shift analysis among chloroplast genome regions indicates that *N. pallida* differs from other *Prosopis* s.l. species in the positions of all boundaries. Yet, these differences were minor, with a maximum displacement of a few hundred bases at the beginning of LSC. Asaf et al. [46] reported similar results when evaluating other *Prosopis* s.l. species. The shifting of boundaries between regions is a primary factor to the variation in chloroplast genome size. This has been observed when comparing closely related species in different groups, and it can be explained by the expansion and contraction of chloroplast regions [102–104]. Nonetheless, these variations are typically minor among related species, resulting in similar lengths of their chloroplast genomes and regions.

We successfully sequenced, assembled and annotated the chloroplast genome of *N. pallida in this study*. Our results allowed us to make comparisons with other species of *Prosopis* s.l., revealing a high degree of similarity with some differences at the structural and genetic level. We also used the sequence produced along with other published chloroplast sequences to perform a phylogenomic analysis that showed that *N. pallida* grouped with the other *Neltuma* species and with *P. cineraria*. Finally, divergence comparisons with other chloroplast genomes of *Neltuma* and *Prosopis* s.l. showed that within the group exists a high level of sequence identity. Nevertheless, certain divergent sequences and genes that could be interesting for the development of molecular markers. The data generated by this research can aid in the development of new lines of research that enhances the understanding of the diversity and preservation of this species in a more effective manner .

## Conclusions

The *Neltuma pallida* chloroplast genome closely resembles those of closely related species. It has a size of 162,381 bp with a classical quadripartite structure and a GC content of 35.97%. The genome contains 132 genes, comprising 85 protein-coding genes, 8 rRNA-coding genes and 39 tRNA-coding genes. The codon usage analysis of the 85 protein-coding genes showed that isoleucine and lysine were the two most prevalent amino acids, and there was a clear preference for codons that have A or T/U in their third position. Also, the repetitive sequence identification enabled us to discover 142 SSR with potential as population-level markers.

Phylogenetic reconstruction revealed that *N. pallida* grouped together with the other species of the genus *Neltuma* and with *P. cineraria*. Additionally, the comparison of the *N. pallida* chloroplast genome with others from close species exhibited a high degree of similarity, particularly in coding regions. These findings can be useful for further diversity or genetic improvement studies in *N. pallida*.

## Methods

### Plant material

Young leaves of *N. pallida* were collected from an adult Algarrobo tree situated in the Bosque de Pómac Historic Sanctuary, Lambayeque Department, Peru (6°26′39.4″ S 79°48′16.6.6″ W). The collection was carried out under the authorization granted by “Resolución Jefatural de Santuario Histórico Bosque de Pómac N° 003-2020-SERNANP-JEF”. The collected samples were transported in paper envelopes for DNA extraction. The species was identified by PhD (c) Marinoli Rivas from the Laboratorio de Gimnospermas y Monocotiledoneas of the Museo de Historia Natural – UNMSM, using the descriptions made by Burkart [1] and Hughes et al. [2]. The voucher is available at the Museo de Historia Natural - UNMSM herbarium (USM N° 335,439) in Lima, Peru.

### DNA extraction and sequencing

For DNA extraction, we used 0.2 g of ground sample in 2% CTAB buffer and followed the protocol published by Doyle [105] with minor modifications. The extracted DNA was purified with a DNAse-free RNAse A treatment at 37 °C for 1 hr. DNA quality and concentration were assessed with Nanodrop™ One C (Thermo Scientific, Massachusetts, USA) and Qubit™ 4 (Invitrogen, Massachusetts, USA), respectively. Additionally, sample integrity was also verified by 1% agarose gel electrophoresis.

DNA sequencing was performed using Illumina methodology by contracting the services of an external laboratory. The TruSeq DNA PCR-Free kit (Illumina Inc., California, USA) was used to construct the sequencing library.

The quality of the generated reads was assessed with FastQC v0.11.9 (https://github.com/s-andrews/FastQC) and then filtered with Trimmomatic v0.39 (https://github.com/usadellab/Trimmomatic) [106].

### De novo assembly of *Neltuma pallida* chloroplast genome

To obtain the sequence of the *N. pallida* chloroplast genome (Genbank: OR178743), a de novo assembly was performed with GetOrganelle v1.7.6.1 (https://github.com/Kinggerm/GetOrganelle) [107]. This is a toolkit that combines Bowtie2 [108], BLAST [109], SPAdes [110], and Python libraries to identify sequences specific to the chloroplast DNA and assemble the chloroplast genome. GetOrganelle parameters were tuned to use the filtered data in the previous step (forward and reverse reads), and to conduct 1000 rounds of extension iterations. Also, we set up GetOrganelle to search sequences corresponding to plant plastids. The other options were left as default. The obtained GFA file was visualized with Bandage v0.9.0 (https://rrwick.github.io/Bandage/) [111] to explore the chloroplast genome structure. The average coverage of the final genome assembly was 130X.

### Genome annotation of *Neltuma pallida* chloroplast genome

Genome annotation by prediction was performed using GeSeq v2.03 [112] in the Chlorobox web server (https://chlorobox.mpimp-golm.mpg.de/), with *N. juliflora* (Genbank: NC049132) taken as reference. Chloroplast Inverted Repeats (IR), *rps*12 interspersed gene, protein-coding sequences, transfer RNAs (tRNAs), and ribosomal RNAs (rRNAs) were all annotated. For proteins and RNAs, 25 and 85% identity were set as thresholds for annotation, respectively. Furthermore, tRNAscan-SE v2.0.7 [113], found on the same server, was used as a secondary tRNA annotator. Additionally, the external annotator Chloë v0.1.0 (https://github.com/ian-small/chloe), which is also found on the web server, was utilized. A manual curation was performed to finish the annotation for each gene, comparing the genes with their homologues found in other chloroplast genomes of species of the genera *Neltuma*, *Prosopis* and *Strombocarpa*.

The sequences of the annotated protein-coding genes were blasted in the KEGG (https://www.kegg.jp/) and UniProt (https://www.uniprot.org/) databases. In this way we sought to perform functional annotation.

### Codon usage of protein-coding sequences

This analysis was solely performed on protein-coding sequences. Codon usage was analyzed by calculating codon frequency and the Relative Synonym Codon Usage (RSCU) values. If the RSCU was greater than 1, this codon was considered to be used more frequently, whereas if the RSCU was less than 1, the opposite was considered to be true. DAMBE5 v7.3.2 (http://dambe.bio.uottawa.ca/index.aspx) [114] was used to perform this analysis.

### Identification of repetitive sequences in the chloroplast genome

Several programs were employed for the identification of repetitive sequences. The identification of tandem repeats, both microsatellite repeats (SSR, Short Sequence Repeat) and long sequences was performed with Phobos v3.3.12 (https://www.ruhr-uni-bochum.de/ecoevo/cm/cm_phobos.htm) [115]. The threshold for determining repeats was a minimum of 10 repeats for mononucleotides, 8 repeats for dinucleotides, 4 repeats for trinucleotides and tetranucleotides, and 3 repeats for pentanucleotides and hexanucleotides.

For the other repetitive sequences: palindromes (P), forward (F), and reverse (R), the RepEX web server (http://bioserver2.physics.iisc.ac.in/RepEx/index.html) [116] was used. Additionally, IUPACpal was used (https://sourceforge.net/projects/iupacpal/) [117] for palindromic sequences, and Vmatch v2.3.1 (http://www.vmatch.de/) [118] for forward repeats. For all of these repeats, a minimum length of 15 bp was considered, and 90% identity with respect to their template as threshold.

### Phylogenomic relationships of *Neltuma pallida*

The *N. pallida* chloroplast genome was aligned with previously published chloroplast genomes of 30 species of the Caesalpinioideae subfamily (Table S7). Sequences were retrieved from the NCBI database (https://www.ncbi.nlm.nih.gov/) and aligned using the MAFFT web server (https://mafft.cbrc.jp/alignment/software/) [119] with default options.

Phylogenetic inference was done with two types of algorithms: Bayesian Inference (BI), in Beast2 v2.7.3 (https://www.beast2.org/) [120], and Maximum Likelihood (ML), in RAxML-HPC2 v8.2.12 [121]. For BI, the GTR + I + G substitution model (Yang 1994) with a Markov Chain Monte Carlo of 1,500,000 steps was used, removing the initial 10% of these. For ML, the GTR + I + G model with 1000 Bootstrap replicates was used. We used jModelTest 2.1.10 v20160303 (https://github.com/ddarriba/jmodeltest2) [122] to determine the substitution models.

### Sequence divergence of chloroplast genomes

The Vista web server (https://genome.lbl.gov/vista/index.shtml) [123], with the mVista function in Shuffle-LAGAN mode was utilized for sequence divergence analysis. This mode enables the detection of rearrangements within genomes. The chloroplast genome sequence of *N. pallida* was used as a reference along with its annotation. The comparison was made with the chloroplast genomes of *N. juliflora*, *P. farcta* (Genbank: NC061227), *S. tamarugo* (Genbank: NC060845) and *A. ligulata* (Genbank: NC026134).

### Genetic distance analysis of coding sequences

Sequences of 74 chloroplast genes from *N. pallida*, *N. juliflora*, *P. farcta*, *S. tamarugo* and *A. ligulata* (Genbank: NC026134) were aligned using ClustalW [124] in Bioedit v7.2.6 [125]. The generated alignments were used to calculate the genetic distance of the sequences of all species from *N. pallida*. The genetic distance was calculated using the p-distance algorithm with Mega X v10.1.8 (https://www.megasoftware.net/) [126].

### Boundary shift analysis between chloroplast genome regions

A comparison was made of the position of boundaries between single copy regions (LSC and SSC) and inverted regions (IRA and IRB) in 5 species of the subfamily Caesalpinioideae: *N. pallida*, *N. juliflora*, *P. farcta*, *S. tamarugo* and *A. ligulata*. These boundaries were called JLB (LSC-IRB boundary), JSB (IRB-SSC), JSA (SSC-IRA) and JLA (IRA-LSC). For the positions of the boundaries, the distance between the boundaries and the genes closest to or over the boundaries was calculated.

### Supplementary Information

**Additional file 1.**

**Additional file 2.**

## Data Availability

The data supporting the findings of this study is freely available in GenBank on the NCBI website (https://www.ncbi.nlm.nih.gov/) using the accession number OR178743, which corresponds to the *Neltuma pallida* chloroplast genome sequence. The reads that were used to assemble the chloroplast genome in this study were deposited at the NCBI Sequence Read Archive (SRA) under accession SRR25007997.

## Abbreviations

BI: Bayesian Inference
F: Forward repeat
IR: Inverted Repeat
JLA: Boundary between LSC and IRA
JLB: Boundary between LSC and IRB
JSA: Boundary between SSC and IRA
JSB: Boundary between SSC and IRB
LSC: Large Single Copy
ML: Maximum Likelihood
ORF: Open Reading Frame
P: Palindrome repeat
R: Reverse repeat
rRNA: Ribosomal RNA
RSCU: Relative Synonymous Codon Usage
SSC: Small Single Copy
SSR: Simple Sequence Repeat or Microsatellite
tRNA: Transfer RNA
