# Supplementary material for: Assembly, annotation and analysis of the chloroplast genome of the Algarrobo tree Neltuma pallida (subfamily: Caesalpinioideae)
Source: BMC Plant Biol. 2023 Nov 16;23:570. doi: 10.1186/s12870-023-04581-5 (PMC10652460; doi:10.1186/s12870-023-04581-5)
Supplement: Supplementary file 1 — Additional file 1. [file 12870_2023_4581_MOESM1_ESM.docx]

**Assembly, annotation and analysis of the chloroplast genome of the Algarrobo tree *Neltuma pallida* (Subfamily: Caesalpinioideae).**

Esteban Caycho^1^, Renato La Torre^1^, Gisella Orjeda^1*^

^1^Laboratory of Genomics and Bioinformatics for Biodiversity, Faculty of Biological Sciences, Universidad Nacional Mayor de San Marcos, Lima 15081, Peru.

^*^Correspondence: [morjedaf@unmsm.edu.pe](mailto:morjedaf@unmsm.edu.pe)


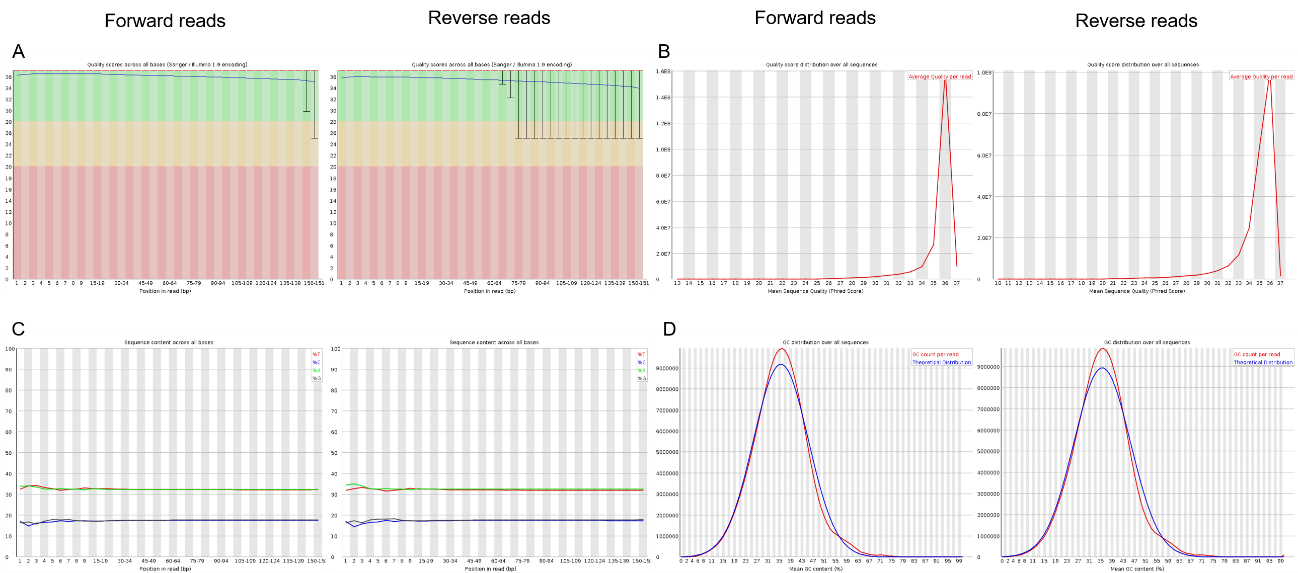


**Figure S1.** Quality analysis of the filtered reads used to assemble the *Neltuma pallida* chloroplast genome. (A) Quality scores across all bases. (B) Quality score distribution over all sequences. (C) Sequence base content across all bases. (D) GC distribution over all sequences.


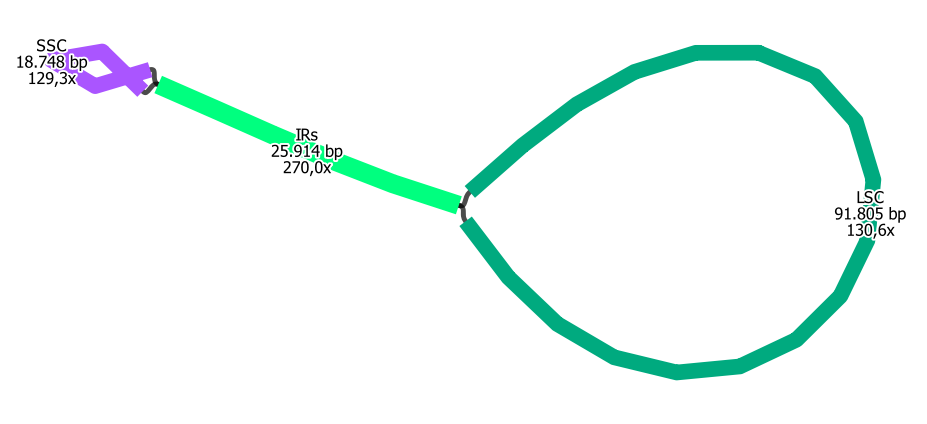


**Figure S2.** Graphical representation of the *N. pallida* chloroplast genome structure. The names, sequence length and sequencing coverage of each region of the genome are displayed over the figure.
